# Rapid-onset hypernatremia induced by central diabetes insipidus leading to osmotic demyelination syndrome: a case report

**DOI:** 10.3389/fmed.2025.1498731

**Published:** 2025-04-30

**Authors:** Yifan Zhang, Ruijun Chen, Xiangxu Kong, Yuexin Yan, Shengyuan Su

**Affiliations:** ^1^Department of Intensive Care Medicine, Shenzhen Bao’an District People’s Hospital, Shenzhen, China; ^2^Department of Neurology, Shenzhen Bao’an People’s Hospital, Shenzhen, China

**Keywords:** hypernatremia, central diabetes insipidus, osmotic demyelination syndrome, case report, sodium correction

## Abstract

This case study describes a middle-aged male patient who developed persistent hypernatremia due to central diabetes insipidus (CDI), presenting with polyuria (up to 8.5 L/24 h), polydipsia, and hypotonic urine (urine specific gravity < 1.005). A positive response to the desmopressin test confirmed the diagnosis of CDI. The excessive loss of body water led to a peak serum sodium level of 195 mmol/L, resulting in Osmotic demyelination syndrome (ODS), clinically manifesting as sluggish responses and symmetrical limb paralysis. The patient was treated with hypotonic fluid replacement combined with desmopressin while ensuring a controlled reduction in serum sodium levels (≤10 mmol/L within 24 h). As a result, as serum sodium and urine output gradually normalized, the patient’s consciousness and limb strength progressively recovered. This case highlights the risk of ODS in patients with severe hypernatremia caused by CDI. A slow and controlled correction of serum sodium levels is crucial in preventing cerebral edema, and early rehabilitation plays a vital role in improving neurological outcomes.

## Introduction

Hypernatremia is a severe electrolyte disturbance in the Intensive Care Unit (ICU), associated with a high mortality rate (40%–60%) (1). Acute hypernatremia can lead to neurological complications, including seizures, hypertonia, and altered consciousness, with serum osmolality likely being the most significant factor (2). A common cause of hypernatremia associated with acute encephalopathy is central diabetes insipidus (CDI), a disorder resulting from either insufficient secretion or impaired action of antidiuretic hormone (ADH). The severity of polyuria correlates with the extent of neuronal damage; complete destruction results in total CDI and severe polyuria, while partial damage with some residual AVP secretion leads to partial CDI with less severe polyuria (3). Diagnosing CDI is notably complex (3). Given its rarity, with an estimated incidence of 1 in 25,000 (4), emphasizing the need for well-defined diagnostic protocols grounded in reliable and accessible research. Advances in laboratory methods have improved the accuracy of these protocols (5), yet the final diagnosis requires a synthesis of clinical, laboratory, and radiological data. Previous case series have reported that 25%–50% of CDI cases are idiopathic (6). In cases where the etiology is unclear, treatment is primarily focused on alleviating symptoms and managing the patient’s fluid and electrolyte balance. The therapeutic aim is to reduce urine output, increase urine concentration, and maintain adequate hydration. Correcting hypernatremia, particularly when it has been present for some time, demands careful balance; overly rapid correction may result in severe, potentially irreversible brain damage. Therefore, dynamic monitoring of serum sodium levels is critical. In most instances, dynamic testing of the AVP–renal axis is necessary. Further imaging, such as MRI of the sella turcica and suprasellar region, is required to determine the underlying cause (7).

Osmotic demyelination syndrome (ODS) is a central nervous system disorder characterized by symmetrical demyelination at the brainstem base without inflammation (8), often linked to chronic alcoholism and disturbances in electrolytes, particularly when hyponatremia is corrected too rapidly (9). The underlying mechanism of ODS involves neuronal dehydration due to rapid osmotic shifts, leading to oligodendrocyte damage and myelin dissolution (10). Though cases of ODS in conjunction with CDI are infrequent, they have been documented, including occurrences of CDI and ODS resulting from lymphocytic hypophysitis (11), as well as renal diabetes insipidus induced by hypokalemia leading to ODS (12). Additionally, there are reports of CDI-related ODS occurring after pregnancy, the postpartum period, and in cases of alcohol-induced hyponatremia (13, 14). In this case report, we present a patient with polyuria due to CDI, who developed ODS as a result of rapid sodium elevation caused by excessive fluid loss without timely rehydration. Through controlled fluid replacement and gradual sodium correction, the patient achieved a favorable outcome.

## Case report

**FIGURE 1:**
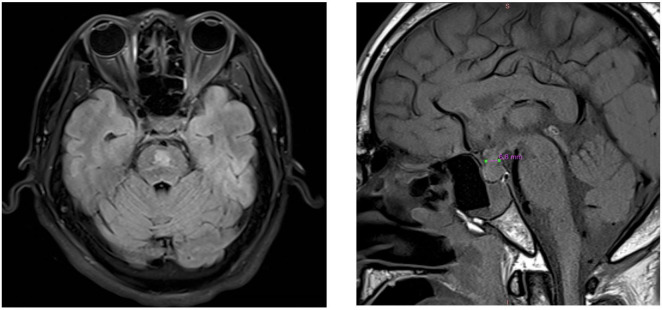
Neuroimaging studies of the patient. **(Left panel)** Presents an axial brain MRI: demyelinating lesions are evident in a symmetrical pattern within the central pons. **(Right panel)** Illustrates a sagittal brain MRI: the pituitary stalk is thickened to 6.8 mm, with the absence of the posterior pituitary bright signal.

**FIGURE 2:**
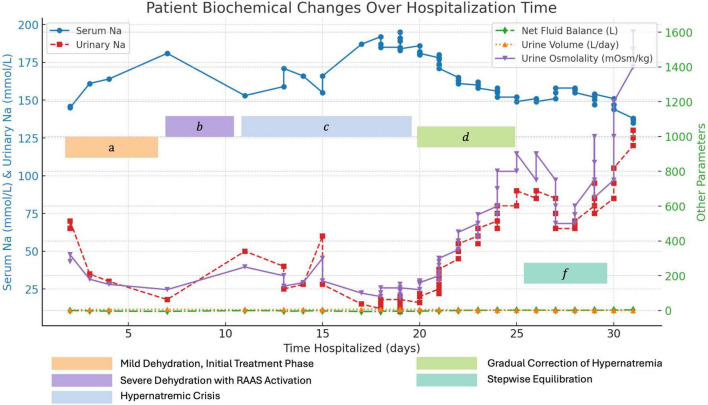
Trends in serum and urinary electrolytes, as well as fluid balance changes during hospitalization.

A 41-year-old male was admitted to our hospital with a 3-month history of dry mouth and polyuria. A water deprivation test demonstrated that urine output did not decrease after fluid restriction but significantly decreased following desmopressin administration, with a corresponding increase in urine osmolality. Plasma ADH levels were found to be low. At that time, his sodium, cortisol, and pituitary-related hormone levels were within normal limits, though his sodium was at the upper end of the normal range (144 mmol/L). His medical history included hypertension and a previous tonsillectomy. Upon admission, he had a fever, and elevated white blood cell count. Clinical and radiological assessments confirmed the presence of a pulmonary abscess. His serum sodium level upon admission was 147.79 mmol/L (reference range: 137–147 mmol/L), while his potassium level was low at 3 mmol/L (reference range: 3.5–4.5 mmol/L). He presented with symptoms of hypotonic polyuria and polydipsia, with significantly increased fluid output, reaching a maximum urine output of 8000 ml per day. A diagnosis of CDI was established made, based on a positive water deprivation-desmopressin test, low urine specific gravity (1.005), urine sodium of 8.2 mmol/24 h (reference range: 130–147 mmol/24 h), and urine potassium of 7.72 mmol/24 h (reference range: 25–100 mmol/24 h). Additionally, MRI revealed the absence of the normal high signal in the posterior pituitary. His sodium levels rapidly rose, increasing by 16 mmol/L within 48 h, peaking at 195 mmol/L. During the first 2 weeks of hospitalization, the patient experienced polyuria without excessive intravenous fluid supplementation, leading to a hypernatremic crisis and increased urine osmolality. Subsequently, the patient developed delirium, slow response, and limb muscle weakness. Brain MRI revealed thickening of the pituitary stalk, blurring of the posterior pituitary signal, and central pontine demyelination (Figure 1), while cerebrospinal fluid (CSF) analysis was unremarkable, confirming the diagnosis of ODS. During the gradual correction of hypernatremia, urine sodium osmolality progressively decreased, and serum sodium levels returned to normal. The patient had no history of lithium use. Figure 2 illustrates the trends in the patient’s serum sodium levels, fluid balance, and osmolality changes. Additionally, the patient developed numerous red papules on the back. However, skin biopsy of the rash revealed no significant pathological findings, ruling out Langerhans cell histiocytosis (LCH). His cortisol levels were mildly elevated at 447.98 ng/ml (reference range: 42.6–248.5 ng/ml), with decreased ADH ( < 1.4 pmol/L), and normal levels of adrenocorticotropic hormone (ACTH), sex hormones, aldosterone, and thyroid hormones (Supplementary Table 1). Immunological tests, including antinuclear antibodies and antineutrophil cytoplasmic antibodies, were negative. The patient’s lung abscess was caused by *Parvimonas micra* infection.

The water deficit was calculated using the formula:

Water deficit = CBW × (140–plasm [Na^+^])^–1^

Where cell body water (CBW) represents total body water, which in young males is typically approximately 60% of lean body weight (15). The patient was administered intravenous infusions of 5% glucose solution and 0.2% sodium chloride solution, along with oral desmopressin (0.05 mg per dose, twice daily) to reduce renal water excretion. The infusion rate was carefully adjusted to ensure that the rate of serum sodium reduction did not exceed 10 mmol/L per 24 h (16). Additionally, acupuncture, massage therapy, and both active and passive rehabilitation exercises were implemented to facilitate neurological recovery and improve limb strength. One month after discharge, follow-up showed resolution of the pulmonary abscess, normalized blood sodium levels, and no remaining neurological deficits. The patient’s pituitary and hypothalamic hormones, while detailed changes in blood sodium, fluid balance, urinary sodium, and urine osmolality are presented in Supplementary Table 2.

## Discussion

Central diabetes insipidus is characterized by a deficiency of ADH, resulting in hypotonic polyuria and polydipsia. This patient presented with persistent polyuria, elevated serum sodium levels, and a positive water deprivation-desmopressin test, leading to a definitive diagnosis of CDI. Given the absence of a history of head trauma, neurosurgical procedures, or lithium use, secondary causes of CDI were considered, including inflammatory, neoplastic, infiltrative, and infectious conditions affecting the hypothalamic-pituitary axis (6). Among these, lymphocytic hypophysitis, LCH, germinoma, and tuberculosis can present with pituitary stalk thickening and loss of posterior pituitary signal on MRI (17). Lymphocytic hypophysitis is an autoimmune disorder characterized by lymphocytic infiltration of the pituitary gland and is frequently associated with other autoimmune diseases. It typically presents with pituitary insufficiency and, while it can lead to CDI, secondary adrenal insufficiency and anterior pituitary hormone deficiencies are also common (18). In the present case, the patient exhibited normal levels of ACTH, thyroid hormones, and sex hormones, with negative autoimmune markers, making lymphocytic hypophysitis an unlikely diagnosis. Furthermore, the patient’s Gutenberg score was −5, indicating a low probability of pituitary carcinoma (19). Another key differential diagnosis is LCH, which commonly manifests as pituitary stalk thickening and CDI, often accompanied by systemic involvement, including skeletal lesions, skin rash, and pulmonary nodules (20). However, a skin biopsy revealed no evidence of Langerhans cell infiltration, with immunohistochemical staining for CD1a and CD20 yielding negative results. Additionally, the patient exhibited no osteolytic bone lesions or hepatosplenomegaly, making LCH an unlikely diagnosis. Germ cell tumors can cause isolated CDI with progressive pituitary stalk thickening and are typically diagnosed based on elevated tumor markers in CSF (21), However, in this case, CSF analysis showed no abnormalities, and no significant lesions were detected in the pineal or suprasellar regions, further minimizing the likelihood of CDI.

A key challenge in this case was severe hypernatremia resulting from excessive free water loss. The patient exhibited polyuria and polydipsia but did not receive intravenous fluid supplementation, leading to a rapid rise in serum sodium due to the excretion of large amounts of hypotonic urine. Consequently, the patient developed neurological symptoms, including delirium, lethargy, and muscle weakness. Hypernatremia induces a hyperosmolar state in brain cells, resulting in cellular dehydration and shrinkage, blood-brain barrier disruption, and endothelial junction impairment (22). ODS is a severe demyelinating disorder typically associated with rapid fluctuations in serum osmolality, usually occurring 2–7 days after electrolyte disturbances (23). While ODS is most commonly linked to the rapid correction of chronic hyponatremia, cases associated with hypernatremia have also been reported (24). The correction of hypernatremia in CDI and ODS must be carefully managed to prevent complications such as cerebral edema and seizures. In patients at risk for ODS, proactive preventive and reactive strategies should be implemented, including the early administration of desmopressin. If overcorrection occurs, serum sodium levels should be reduced using D5W. Although there is no clear evidence supporting the benefit of osmolar reduction beyond 24 h after ODS symptom onset, clinicians may consider attempting osmolar reduction in such cases. Guidelines recommend that the reduction in serum sodium should not exceed 12 mEq/L within the first 24 h of hypernatremia correction, with sodium levels monitored every 2–4 h. If seizures occur, hypotonic fluid administration should be discontinued (25).

For patients with severe neurological impairment, supportive therapy remains essential for survival. Additionally, immunomodulatory treatments, including thyrotropin-releasing hormone, intravenous immunoglobulin, plasmapheresis, and corticosteroids, have been proposed as potential interventions for ODS (26, 27). While early induction of hyponatremia has shown benefits in animal models of ODS, clinical evidence remains limited (28). Furthermore, studies suggest that minocycline may exert anti-inflammatory and anti-apoptotic effects in preventing the progression of acute sodium fluctuations to ODS (29). The pathophysiology of ODS may be linked to glial cell apoptosis and severe blood-brain barrier disruption, with a reported long-term neurological mortality rate of approximately 31% (30). In this case, the patient’s favorable prognosis highlights the potential reversibility of acute ODS with timely sodium correction. Long-term follow-up is crucial, as CDI patients require continuous monitoring of pituitary function, electrolyte balance, and potential complications. The prognosis of ODS is associated with meticulous electrolyte and fluid management, as well as timely imaging surveillance, with studies indicating favorable outcomes for up to 4 years post-diagnosis (31). As of the latest follow-up, the patient’s urine output and serum sodium levels remain within the normal range.

This case underscores the importance of identifying the etiology of CDI in the context of hypernatremia and highlights the risks associated with sodium imbalance. Despite controlled sodium correction, the patient developed ODS, raising concerns about the brain’s vulnerability to osmotic fluctuations in a hypernatremic state. The successful resolution of CDI and neurological complications through appropriate fluid management and rehabilitation emphasizes the necessity of a multidisciplinary approach in managing such complex cases. Further research into optimal treatment strategies for CDI and hypernatremia may contribute to improved long-term outcomes and the minimization of neurological risks.

## Conclusion

This case highlights the severe challenges associated with hypernatremia in CDI due to fluid loss, particularly under hyperosmolar conditions. Early recognition and correction of ODS are crucial for prognosis. The case underscores the importance of cautiously correcting sodium levels, as even controlled reduction of hypernatremia may not entirely prevent neurological complications. A multidisciplinary management approach facilitated the patient’s recovery, incorporating meticulous fluid and electrolyte correction, desmopressin administration, and rehabilitative therapy.

## Data Availability

The original contributions presented in this study are included in this article/Supplementary material, further inquiries can be directed to the corresponding author.

## References

[1] Lamichhane R Yaqub A. Abstract #1408214: Severe hypernatremia due to central diabetes insipidus with adipsia: Challenges in management. *Endocrine Pract.* (2023) 29:S81–2. 10.1016/j.eprac.2023.03.183

[2] Morris-Jones P Houston I Evans R. Prognosis of the neurological complications of acute hypernatraemia. *Lancet.* (1967) 2:1385–9. 10.1016/s0140-6736(67)93022-x 4170055

[3] Kiess W Werther G. Best practice; Research clinical endocrinology & metabolism. Hormone replacement strategies in paediatric and adolescent endocrine disorders. Preface. *Best Pract Res Clin Endocrinol Metab.* (2015) 29:313–4. 10.1016/j.beem.2015.04.012 26051292

[4] Christ-Crain M Bichet D Fenske W Goldman M Rittig S Verbalis J Diabetes insipidus. *Nat Rev Dis Primers.* (2019) 5:54. 10.1038/s41572-019-0103-2 31395885

[5] Fenske W Refardt J Chifu I Schnyder I Winzeler B Drummond J A copeptin-based approach in the diagnosis of diabetes insipidus. *N Engl J Med.* (2018) 379:428–39. 10.1056/NEJMoa1803760 30067922

[6] Chehaider C Oueslati I Mabrouk M Terzi A Yazidi M Chihaoui M. Diabète insipide central: Particularités cliniques et étiologiques. *Annales d’Endocrinologie.* (2023) 84:574. 10.1016/j.ando.2023.07.182

[7] Garrahy A Thompson C. Management of central diabetes insipidus. *Best Pract Res Clin Endocrinol Metab.* (2020) 34:101385. 10.1016/j.beem.2020.101385 32169331

[8] Adams R Victor M Mancall E. Central pontine myelinolysis: A hitherto undescribed disease occurring in alcoholic and malnourished patients. *AMA Arch Neurol Psychiatry.* (1959) 81:154–72. 13616772

[9] Kallakatta R Radhakrishnan A Fayaz R Unnikrishnan J Kesavadas C Sarma S. Clinical and functional outcome and factors predicting prognosis in osmotic demyelination syndrome (central pontine and/or extrapontine myelinolysis) in 25 patients. *J Neurol Neurosurg Psychiatry.* (2011) 82:326–31. 10.1136/jnnp.2009.201764 20826870

[10] Martin R. Central pontine and extrapontine myelinolysis: The osmotic demyelination syndromes. *J Neurol Neurosurg Psychiatry.* (2004) 75:iii22–8. 10.1136/jnnp.2004.045906 15316041 PMC1765665

[11] Chang L Harrington D Milkotic A Swerdloff R Wang C. Unusual occurrence of extrapontine myelinolysis associated with acute severe hypernatraemia caused by central diabetes insipidus. *Clin Endocrinol (Oxf).* (2005) 63:233–5. 10.1111/j.1365-2265.2005.02319.x 16060921

[12] Davenport C Liew A Vic Lau P Smith D Thompson C Kearns G Central pontine myelinolysis secondary to hypokalaemic nephrogenic diabetes insipidus. *Ann Clin Biochem.* (2010) 47:86–9. 10.1258/acb.2009.009094 19940203

[13] Yadav A Sherpali A Bashyal B Kala Kharel K Parajuli N. Osmotic demyelination syndrome with transient diabetes insipidus in postpartum female: A case report. *Ann Med Surg (Lond).* (2023) 85:4096–9. 10.1097/MS9.0000000000000987 37554876 PMC10406002

[14] Bickelhaupt B Neeley J. Poster 365 traumatic brain injury resulting from pontine and extrapontine myelinolysis due to acute onset of pregnancy induced diabetes insipidus: A case report. *PM R.* (2016) 8:S280. 10.1016/j.pmrj.2016.07.292 27673118

[15] Kim S. Hypernatemia : Successful treatment. *Electrolyte Blood Press.* (2006) 4:66–71. 10.5049/EBP.2006.4.2.66 24459489 PMC3894528

[16] Tomkins M Lawless S Martin-Grace J Sherlock M Thompson C. Diagnosis and management of central diabetes insipidus in adults. *J Clin Endocrinol Metab.* (2022) 107:2701–15. 10.1210/clinem/dgac381 35771962 PMC9516129

[17] Kim D Kim P Jung A Choi J Cho Y Yoon H Neoplastic etiology and natural course of pituitary stalk thickening. *J Clin Endocrinol Metab.* (2022) 107:563–74. 10.1210/clinem/dgab732 34614160

[18] Kolsi F Cherif I Khrifech M Hachicha A Jarraya F Boudawara M. Hypophysite lymphocytaire: diagnostic différentiel difficile avec les adénomes hypophysaires. *Neurochirurgie.* (2020) 66:320–1. 10.1016/j.neuchi.2020.06.100

[19] Gutenberg A Larsen J Lupi I Rohde V Caturegli P. A radiologic score to distinguish autoimmune hypophysitis from nonsecreting pituitary adenoma preoperatively. *AJNR Am J Neuroradiol*. (2009) 30:1766–72. 10.3174/ajnr.A1714 19628625 PMC7051493

[20] Rodriguez-Galindo C Allen C. Langerhans cell histiocytosis. *Blood.* (2020) 135:1319–31. 10.1182/blood.2019000934 32106306

[21] Guedes B Souza M Barbosa B Frassetto F Lucato L Ono C Intracranial germinoma causing cerebral haemiatrophy and hypopituitarism. *Pract Neurol.* (2018) 18:306–10. 10.1136/practneurol-2017-001771 29378909

[22] Shah M Mandayam S Adrogué H. Osmotic demyelination unrelated to hyponatremia. *Am J Kidney Dis.* (2018) 71:436–40. 10.1053/j.ajkd.2017.10.010 29277507

[23] Laureno R Karp B. Myelinolysis after correction of hyponatremia. *Ann Intern Med.* (1997) 126:57–62. 10.7326/0003-4819-126-1-199701010-00008 8992924

[24] Beraldo D Duarte S Santos R Mendes C Silveira M Neto A Pontine myelinolysis caused by hypovolemic hypernatremia. *Case Rep Nephrol.* (2020) 2020:4079098. 10.1155/2020/4079098 32963856 PMC7495154

[25] Baldeweg S Ball S Brooke A Gleeson H Levy M Prentice M society for endocrinology clinical guidance: Inpatient management of cranial diabetes insipidus. *Endocr Connect.* (2018) 7:G8–11. 10.1530/EC-18-0154 29930026 PMC6013691

[26] Kalampokini S Artemiadis A Zis P Hadjihannas L Parpas G Kyrri A Osmotic demyelination syndrome improving after immune-modulating treatment: case report and literature review. *Clin Neurol Neurosurg.* (2021) 208:106811. 10.1016/j.clineuro.2021.106811 34358802

[27] Atchaneeyasakul K Tipirneni A Gloria S Berry A Shah K Yavagal D. Osmotic demyelination syndrome: Plasmapheresis versus intravenous immunoglobulin? *Intern Emerg Med.* (2017) 12:123–6. 10.1007/s11739-016-1452-4 27091143

[28] MacMillan T Cavalcanti R. Outcomes in severe hyponatremia treated with and without desmopressin. *Am J Med.* (2018) 131: 317.e1–10. 10.1016/j.amjmed.2017.09.048 29061503

[29] Takagi H Sugimura Y Suzuki H Iwama S Izumida H Fujisawa H Minocycline prevents osmotic demyelination associated with aquaresis. *Kidney Int.* (2014) 86:954–64. 10.1038/ki.2014.119 24759153

[30] Louis G Megarbane B Lavoué S Lassalle V Argaud L Poussel J Long-term outcome of patients hospitalized in intensive care units with central or extrapontine myelinolysis*. *Crit Care Med.* (2012) 40:970–2. 10.1097/CCM.0b013e318236f152 22036854

[31] Lambeck J Hieber M Dreßing A Niesen W. Central pontine myelinosis and osmotic demyelination syndrome. *Dtsch Arztebl Int.* (2019) 116:600–6. 10.3238/arztebl.2019.0600 31587708 PMC6804268

