# Supplementary material for: Rapid-onset hypernatremia induced by central diabetes insipidus leading to osmotic demyelination syndrome: a case report
Source: Front Med (Lausanne). 2025 Apr 30;12:1498731. doi: 10.3389/fmed.2025.1498731 (PMC12075111; doi:10.3389/fmed.2025.1498731)
Supplement: Supplementary file 1 [file Table_1.docx]

**Supplemental Table 1.** Pituitary and adrenal cortex hormone levels in patients, ACTH (Adrenocorticotropic Hormone)

|  | Values |  | Reference |  |
| --- | --- | --- | --- | --- |
| **Hypothalamus** |  |  |  |  |
| Antidiuretic hormone (ADH) | <1.4 | Pmol/L | 1.4-5.6 |  |
| **Anterior Pituitary** |  |  |  |  |
| Thyroid-Stimulating Hormone* | 0.947 | μlU/mL | 0.56--5.91 |  |
| ACTH | 44.7 | pg /ml | 7.2-63.4 |  |
| **Posterior Pituitary** |  |  |  |  |
| Corticosteroids |  |  |  |  |
| Cortisol | | 447.98 | ng/ml | 42.6～248.5 |
| **Sex Hormones** |  |  |  |  |
| Testosterone | 73.23 | ng/dl | 175-781 |  |
| Estradiol | 31 | pg/ml | 0-39 |  |
| Luteinizing Hormone | 1.44 | mIU/mL | 1.24-8.62 |  |
| Follicle-Stimulating Hormone | 2.32 | mIU/mL | 1.27-19.26 |  |
| Aldosterone | 100.8 | pg /ml | 10-160 |  |
|  |  |  |  |  |
