# Supplementary material for: Rapid-onset hypernatremia induced by central diabetes insipidus leading to osmotic demyelination syndrome: a case report
Source: Front Med (Lausanne). 2025 Apr 30;12:1498731. doi: 10.3389/fmed.2025.1498731 (PMC12075111; doi:10.3389/fmed.2025.1498731)
Supplement: Supplementary file 2 [file Table_2.docx]

**Supplemental Table 2.** Results of blood and urinary electrolytes, as well as fluid balance monitoring during hospitalization.

| **TimeHospitalized time (d)/day** | **Serum Na (mmol/L)** | **Urinary Na (mmol/L)** | **Net Fluid Balance (L)** | **Urine Volume (L/day)** | **Urine Osmolality (mOsm/kg)** |
| --- | --- | --- | --- | --- | --- |
| 2 | 146 | 65 | -0.5 | 3.5 | 280 |
| 2 | 145 | 70 | 0 | 3.2 | 320 |
| 3 | 161 | 35 | -2.2 | 5 | 180 |
| 4 | 164 | 30 | -3.5 | 6 | 150 |
| 7 | 181 | 18 | -5 | 7 | 120 |
| 11 | 153 | 50 | -0.5 | 4.5 | 250 |
| 13 | 159 | 40 | -1.5 | 5 | 200 |
| 13 | 171 | 25 | -4 | 6.5 | 140 |
| 14 | 166 | 28 | -3 | 6 | 160 |
| 15 | 155 | 60 | 0 | 4 | 300 |
| 15 | 166 | 28 | -2.5 | 5.5 | 170 |
| 17 | 187 | 15 | -5.5 | 7.5 | 100 |
| 18 | 192 | 12 | -6 | 8 | 80 |
| 18 | 187 | 15 | -5.5 | 7.5 | 100 |
| 18 | 185 | 18 | -4 | 6.5 | 130 |
| 19 | 185 | 18 | -4 | 6.5 | 130 |
| 19 | 183 | 20 | -3.5 | 6 | 150 |
| 19 | 191 | 13 | -5.8 | 7.8 | 90 |
| 19 | 195 | 10 | -6.5 | 8.5 | 70 |
| 19 | 195 | 10 | -6.5 | 8.5 | 70 |
| 19 | 191 | 13 | -5.8 | 7.8 | 90 |
| 19 | 189 | 15 | -5 | 7 | 110 |
| 19 | 184 | 18 | -4 | 6.5 | 130 |
| 20 | 186 | 16 | -4.5 | 6.8 | 120 |
| 20 | 182 | 20 | -3.5 | 6 | 150 |
| 20 | 180 | 22 | -3 | 5.5 | 170 |
| 20 | 181 | 20 | -3.2 | 5.8 | 160 |
| 20 | 181 | 20 | -3.2 | 5.8 | 160 |
| 20 | 180 | 22 | -3 | 5.5 | 170 |
| 20 | 182 | 20 | -3.2 | 5.8 | 160 |
| 20 | 181 | 20 | -3.2 | 5.8 | 160 |
| 21 | 178 | 25 | -2.5 | 5 | 200 |
| 21 | 177 | 28 | -2 | 4.8 | 220 |
| 21 | 180 | 22 | -3 | 5.5 | 170 |
| 21 | 173 | 35 | -1.5 | 4 | 280 |
| 21 | 174 | 32 | -1.8 | 4.3 | 260 |
| 21 | 171 | 38 | -1 | 3.8 | 300 |
| 22 | 165 | 45 | -0.5 | 3.5 | 350 |
| 22 | 163 | 50 | 0 | 3 | 400 |
| 22 | 163 | 50 | 0 | 3 | 400 |
| 22 | 161 | 55 | 0.5 | 2.5 | 450 |
| 23 | 160 | 60 | 0.8 | 2.2 | 500 |
| 23 | 162 | 55 | 0.3 | 2.7 | 420 |
| 23 | 158 | 65 | 1 | 2 | 550 |
| 24 | 156 | 70 | 1.2 | 1.8 | 600 |
| 24 | 158 | 65 | 1 | 2 | 550 |
| 24 | 155 | 75 | 1.5 | 1.5 | 700 |
| 24 | 152 | 80 | 1.8 | 1.2 | 800 |
| 25 | 152 | 80 | 1.8 | 1.2 | 800 |
| 25 | 152 | 80 | 1.8 | 1.2 | 800 |
| 25 | 149 | 90 | 2 | 1 | 900 |
| 26 | 151 | 85 | 1.5 | 1.5 | 750 |
| 26 | 149 | 90 | 2 | 1 | 900 |
| 26 | 149 | 90 | 2 | 1 | 900 |
| 27 | 151 | 85 | 1.5 | 1.5 | 750 |
| 27 | 155 | 75 | 1 | 2 | 600 |
| 27 | 158 | 65 | 0.5 | 2.5 | 500 |
| 28 | 158 | 65 | 0.5 | 2.5 | 500 |
| 28 | 157 | 68 | 0.8 | 2.2 | 550 |
| 28 | 157 | 68 | 0.8 | 2.2 | 550 |
| 28 | 155 | 70 | 1 | 2 | 600 |
| 29 | 152 | 80 | 1.5 | 1.5 | 750 |
| 29 | 150 | 85 | 2 | 1 | 850 |
| 29 | 147 | 95 | 2.5 | 0.5 | 1000 |
| 29 | 154 | 75 | 1.2 | 1.8 | 650 |
| 30 | 151 | 85 | 1.5 | 1.5 | 750 |
| 30 | 147 | 95 | 2.5 | 0.5 | 1000 |
| 30 | 144 | 105 | 3 | 0.2 | 1200 |
| 31 | 138 | 120 | 3.5 | 0.1 | 1400 |
| 31 | 137 | 125 | 3.8 | 0 | 1500 |
| 31 | 135 | 130 | 4 | 0 | 1600 |
